# Exploring peer education for migrant informal caregivers of mentally ill loved ones: a realist evaluation protocol

**DOI:** 10.3389/fpubh.2025.1623903

**Published:** 2025-08-13

**Authors:** Malin Hollaar, Paul Kocken, Özgül Uysal-Bozkir, Maria Smedts, Semiha Denktaş

**Affiliations:** ^1^Erasmus School of Social and Behavioural Sciences, Erasmus University Rotterdam, Rotterdam, Netherlands; ^2^Indigo Preventie Rijnmond, Rotterdam, Netherlands

**Keywords:** informal caregivers, migration background, mental illness, mental health, peer education, integrated care, realist evaluation

## Abstract

**Introduction:**

Informal caregivers with a migration background who care for someone with a mental illness often experience elevated caregiver burdens due to factors such as discrimination, language barriers, and stigma. In The Netherlands, a peer education intervention called ‘They Are not Mental?!’ (TANM) addresses these challenges by reducing stigma, increasing help-seeking behaviors, and improving access to healthcare. This transdisciplinary study evaluates how contextual factors and mechanisms influence its outcomes.

**Description:**

This research protocol outlines a realist evaluation of the intervention using a mixed-methods design, including interviews, post-questionnaires, and observations. The study will develop and refine the program theory to determine for whom, in what contexts, why, and how the intervention works.

**Discussion:**

This protocol shows how we plan to investigate how, why, for whom and under what circumstances TANM produces its intended outcomes, using a realist evaluation approach. This approach is well-suited for evaluating complex interventions because it accommodates for dynamic and iterative interventions. Its focus is on understanding patterns and mechanisms within specific contexts, using program theories.

**Conclusion:**

The evaluation of TANM will inform future intervention adaptations and guide future efforts to support vulnerable populations, particularly migrants navigating caregiving challenges.

## 1 Introduction

Informal caregivers – often family members or friends who provide unpaid care to loved ones – play an important role in supporting individuals with a diagnosis of a mental health condition (1, 2). For caregivers with a migration background, meaning those who have moved to a new country and are in a situation in which they might be less familiar with local customs, norms, or care system, this responsibility often comes with additional challenges and increased stress, due to stigmatizing beliefs regarding mental illness, language and cultural barriers and limited access to support services (3–5). These caregivers face a “double adaptation burden,” with stress intensified by their caregiving role and experiences as migrants. To alleviate this burden, providing appropriate support is important (5, 6). In response, the culturally sensitive intervention “Ze Zijn Toch Niet Gek?!” (translation: “They Are not Mental”; hereafter TANM) was developed to support informal caregivers with a migration background who care for a loved one with a mental illness. This paper presents the protocol for a planned realist evaluation of TANM, aiming to develop a theoretical framework which explains the mechanisms through which support programs like TANM produce their desired outcomes and gain insights for its improvement.

Members of migrant communities are often at increased psychological health risks, due to challenges such as adapting to a new culture and encountering discrimination. As such, informal caregivers play an important role in supporting those affected (4, 7, 8). Caregiving for someone with a mental illness can be stressful and difficult for all informal caregivers, irrespective of migration status, but caregivers with a migration background face additional challenges that can intensify this caregiver burden (1, 3, 9, 10). These challenges include stigma, which is a general phenomenon across societies and can be influenced by culture-specific explanatory models – such as viewing it as a result of black magic, spirits, punishment of a divine entity or higher power, or the evil eye. Stigma can foster feelings of shame within families and discourage both patients and caregivers from seeking help (3, 4, 11–15). Additionally, cultural expectations around family caregiving, including gendered norms and ideas about family obligations, can further increase caregiver burden (3, 16, 17).

These cultural taboos, combined with systemic barriers like language differences and cultural misunderstandings, can make it difficult for informal caregivers and their loved ones to acknowledge the need for support, limiting the access to formal care (5). These intersecting challenges make caregiving for individuals with mental illness particularly difficult within migrant communities.

One way to address barriers in accessibility is through integrated care, which focuses on breaking down organizational boundaries and aligning services to better meet the needs of individuals, rather than being shaped by what each organization provides separately (18). Integrated care requires collaboration between healthcare, social and community services (18, 19), and ideally, the involvement of the target group. By making care systems more navigable and tailored to the target group’s needs, integrated care is a promising way to empower vulnerable individuals (20). This is particularly relevant for informal caregivers with a migration background, who often face additional challenges in navigating healthcare systems and accessing support (5). For caregivers of individuals with mental illnesses, tailored interventions are important for addressing their physical, psychological, and social needs. Through collaboration with relevant services, integrated care can better meet these needs and enhance the overall quality of psychosocial care by supporting these informal caregivers, who may experience additional barriers due to language, cultural differences, or unfamiliarity with available services (5, 21–23).

To address the challenges faced by informal caregivers with a migration background who care for a loved one with a suspected mental illness, TANM was developed as a peer education program. A key aim of TANM is to enhances caregivers’ resilience – the dynamic process of adapting to adversity, which helps buffer against caregiver burden (24–26) - through improved access to information, reduced stigma surrounding mental health, and stronger connections between migrant families and the healthcare system. Additionally, TANM enhances coordination between community-based support and healthcare organizations, by incorporating elements of integrated care. A detailed description of the TANM, including its structure and implementation, is provided in Section 2.

Since its development in 2014, TANM has undergone slight adjustments, including changes in funding and target groups. Its evolving nature requires an evaluation method that can adapt to these changes. A realist evaluation (RE) design was chosen because it provides a framework not only to assess whether the intervention works, but also to explore why, for whom, and under what circumstances it works. This approach is well-suited for an exploration to understand how underlying contexts and mechanisms – such as stigma and taboos – impact the intended outcomes of TANM (27). Given the complexity of these factors, this research takes a transdisciplinary approach, involving collaboration with the intervention’s coordinating organizations to ensure that different perspectives are integrated into the evaluation process circumstances (27–29). This research aims to evaluate TANM using a RE approach, in order to develop a program theory that explores how contexts and mechanisms, such as stigma and taboos, impact its intended outcomes. The research will answer the following questions:

*‘Did intervention participants, ambassadors and coordinators perceive changes in its short-term outcomes related to discussing mental health taboos, understanding mental illnesses and caregiver support, and their perception of the healthcare sector? How did the intervention components and contextual factors contribute to this change?’*

Considering the goals of TANM, we expect these changes to have a positive direction; that is, we anticipate that participants will discuss mental health taboos with their friends and families, develop a better understanding of mental illnesses and caregiver support, and hold a more positive perception of the healthcare sector.

In the following sections, we describe key components of our realist evaluation design, including the program theory, data collection methods, and planned analyses.

## 2 Intervention description

TANM is a peer-facilitated intervention aimed at strengthening the resilience of informal caregivers with a migration background who support a loved one with a suspected mental illness in the second largest city of the Netherlands, Rotterdam. Developed collaboratively by a community empowerment organization, a mental health prevention agency and a support association for families affected by mental health issues, TANM is a 3-session program that aims to increase resilience in the long-term, by (1) addressing mental health taboos (2); improving access to information and care and (3) fostering trust between migrant communities and the healthcare sector. Since the long-term goal of increasing caregivers’ resilience extends beyond the scope of the current research, we will focus on evaluating its short-term objectives, which include: encouraging open discussions about mental health within migrant communities, improving understanding of mental illnesses and available support for caregivers, and fostering trust between migrant families and the healthcare sector to reduce barriers to professional support.

TANM relies on peer educators, referred to as *ambassadors*, who share similar lived experiences with the participants, particularly through their migration background and, for some ambassadors, their caregiving role. Peer education is an approach to engage ‘hard-to-reach’ populations, such as those with limited resources, language barriers or distrust in the healthcare system. Peers serve as intermediaries who can leverage cultural understandings and trust to identify participants and adapt the intervention’s messages to meet the group’s needs (30–32).

Within TANM, ambassadors are volunteers who are active community members, often demonstrated through a strong social network or prior volunteer experience in their neighborhoods, and they recognize the importance of discussing mental health. Ambassadors are invited through outreach by the community empowerment organization, as well as through referrals from ambassadors of previous years. They are selected based on the following criteria:

- Affinity with and access to target groups;
- Willingness to share personal experiences;
- Ability to lead discussions and educate others;
- Ability to establish contact with healthcare professionals and translate the needs and concerns of the target group to these professionals.

Once selected, ambassadors receive specialized training, covering three main areas: (1) basic knowledge of mental health conditions, such as depression, psychosis and schizophrenia, which includes symptoms, causes, treatment options and the effects of medication and substance use; (2) informal caregiving, including definitions, perceptions, signs of caregiver burden, and available support services; and (3) practical skills in peer education, such as presentation techniques and an introduction of the content of the peer education sessions. The training is delivered in the Dutch language by a prevention specialist with a background in psychology, a Family Experience Expert (FEE; explained in Section 2.1 about Phase 3) and a caregiver support coach affiliated with the local caregiver support service, which has been part of TANMs coordinating organizations since 2023. The training is highly interactive and fosters a culturally sensitive approach by encouraging ambassadors to share personal experiences and explanatory models they are familiar with. A key message throughout the training is that differing perspectives on mental health conditions can coexist, and that creating space for these perspectives during the peer education sessions is essential.

After completing the training, ambassadors recruit participants for their groups and lead the intervention’s peer education sessions in the dominant language spoken within their group. This could be Dutch, but also, for example, Arabic or Turkish. Ongoing support is provided to the ambassadors in two ways, through regular feedback meetings with members of the coordinating organizations and fellow ambassadors, and through direct access to a designated contact person within the coordinating organizations who is available for individual questions, support or guidance through phone, messaging or in-person contact.

Our evaluation will focus on the current round of TANM (September 2024 – March 2025), which is co-organized by four partners, following the addition of a helpline and support service for caregivers in Rotterdam in 2023.

### 2.1 Intervention structure and content

**Figure 1:**
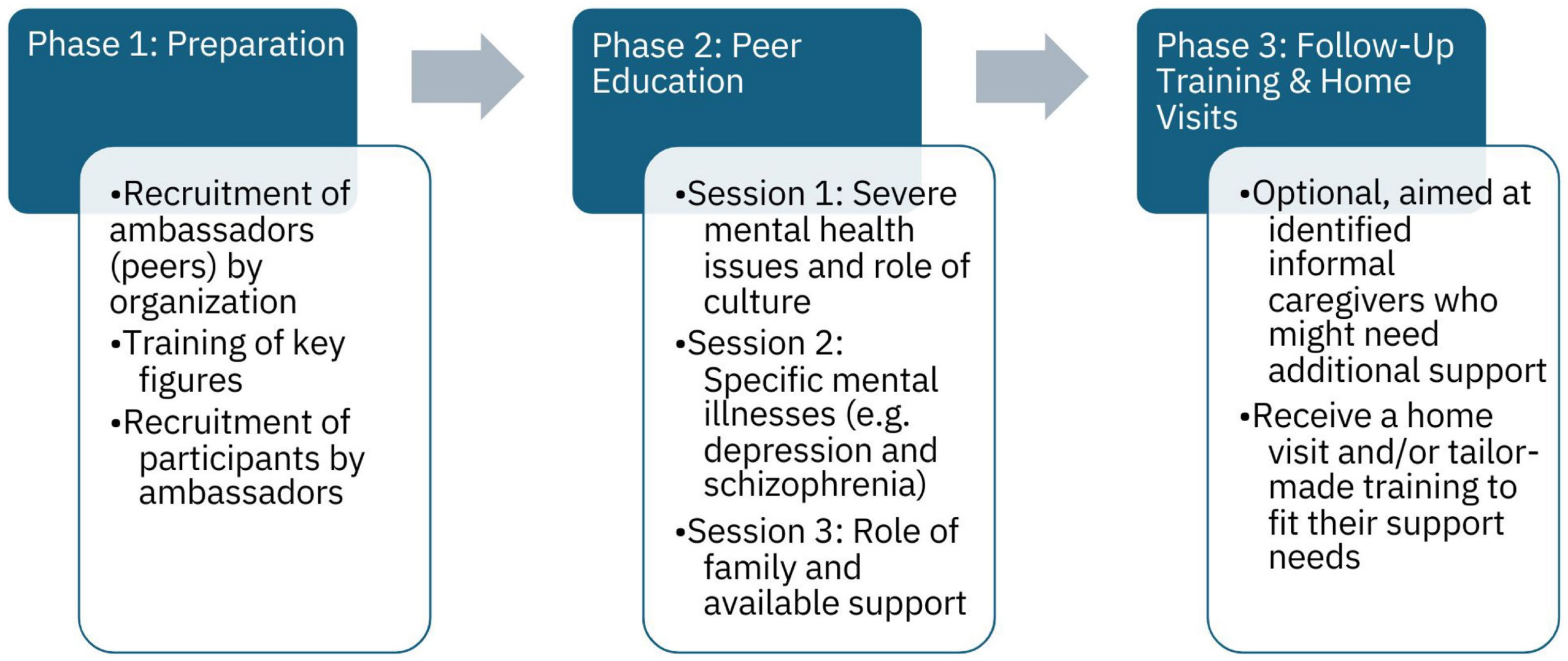
Phases of the intervention TANM.

The intervention is structured into three main phases (see Figure 1 for an overview): the preparation phase, the peer education sessions, and the follow-up training and home visits phase.

#### 2.1.1 Phase 1 – preparation

The organization of TANM recruits ambassadors – i.e. key figures from various communities in Rotterdam. Ambassadors undergo training to become peer educators. This training equips them with the knowledge and skills needed to identify symptoms of mental illness, facilitate peer education sessions, and recruit participants for Phase 2.

#### 2.1.2 Phase 2 - peer education sessions

Peer educators facilitate three structured sessions, focusing on topics such as understanding mental health issues, the role and challenges of informal caregiving, and available support options. Detailed session topics and objectives are outlined in Appendix I. Ambassadors identify participating informal caregivers who may need additional professional support and refer them Phase 3.

#### 2.1.3 Phase 3 - follow-up training and home visits

This optional phase provides tailored support to meet the needs of the identified informal caregivers. For each group, a member of one of TANM’s coordinating organizations, together with the ambassador, conducts a needs assessment to determine the most appropriate follow-up support for the identified caregivers. This may include five to six training sessions with a psychologist for a group of identified informal caregivers, which are based on the training ‘Psychological vulnerability in your immediate environment’ (33) but adapted to fit the unique needs of migrant caregivers. Alternatively, one-on-one consultations by a Family Experience Expert (FEE) for an individual caregiver may be offered. The FEE is someone who has personal experience supporting a relative with mental health issues and has received specialized training, enabling them to reflect on their own experiences, develop communication skills, and establish professional boundaries. Phase 3 is flexible and not attended by all Phase 2 participants.

## 3 Methods

Realist evaluation (RE) is a theory-driven approach that provides a framework for evaluating interventions, policies, measures, and other complex social programs or systems. It examines what works, how, for whom, why and under which circumstances, considering the influence of factors like culture and socioeconomic status on outcomes (27, 28, 34, 35). The underlying idea behind REs is that outcomes are the product of multiple causes and depend on mechanisms and contextual elements (36, 37). Together, these form context-mechanism-outcome (CMO) configurations, which form the basis of the so-called program theory (37). There is no standard methodology for REs, but it is encouraged to use a mixed-methods approach, combining both quantitative and qualitative methods (35, 38). This flexibility makes RE particularly suited for evaluating complex interventions, like TANM (27, 28, 35). Our RE of TANM consists of both qualitative and quantitative methods and will follow the RE reporting standards as made clear by the RAMESES-II project (37).

**Figure 2:**
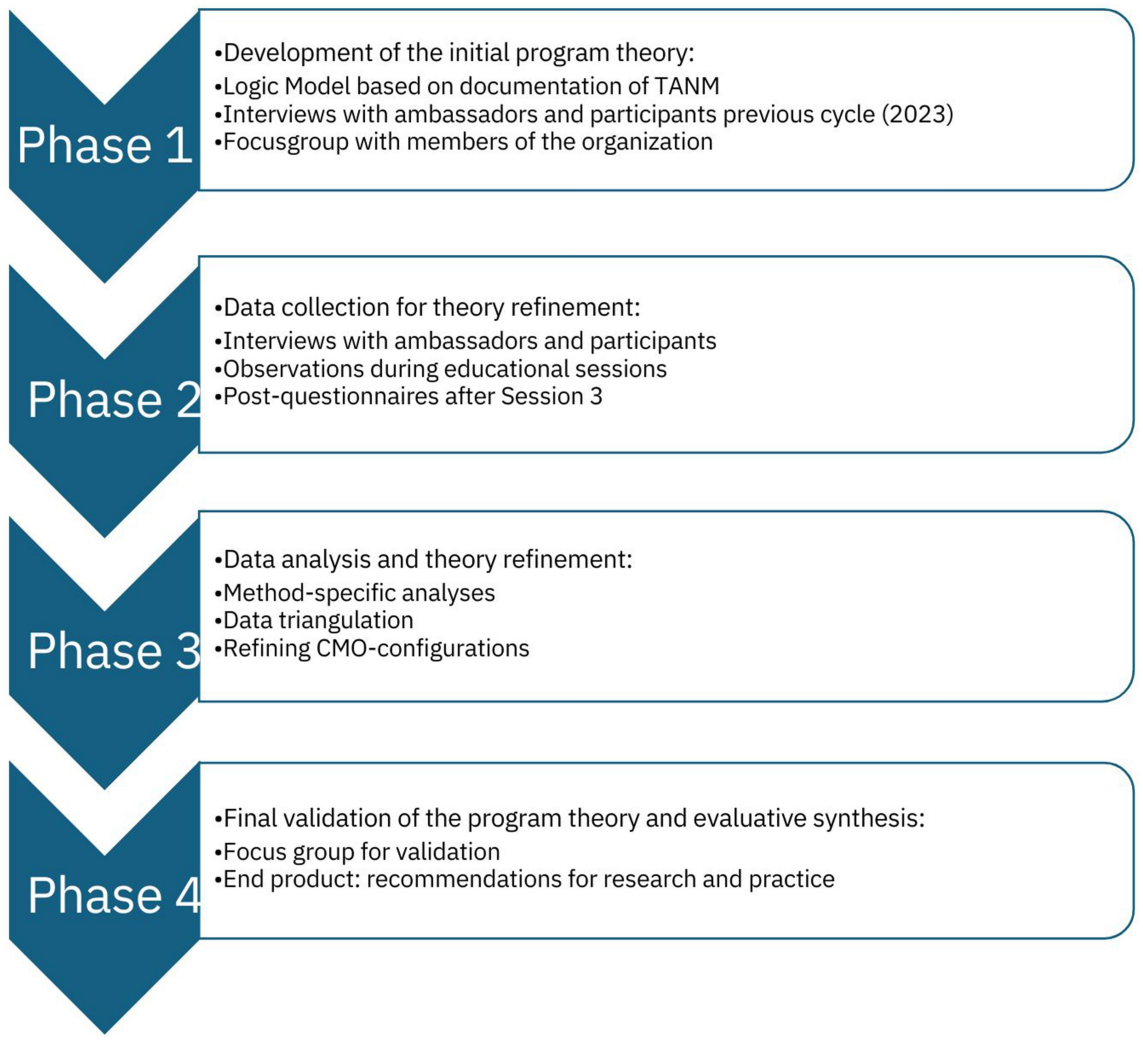
Four phases and corresponding methodologies of our RE approach.

**Figure 3:**
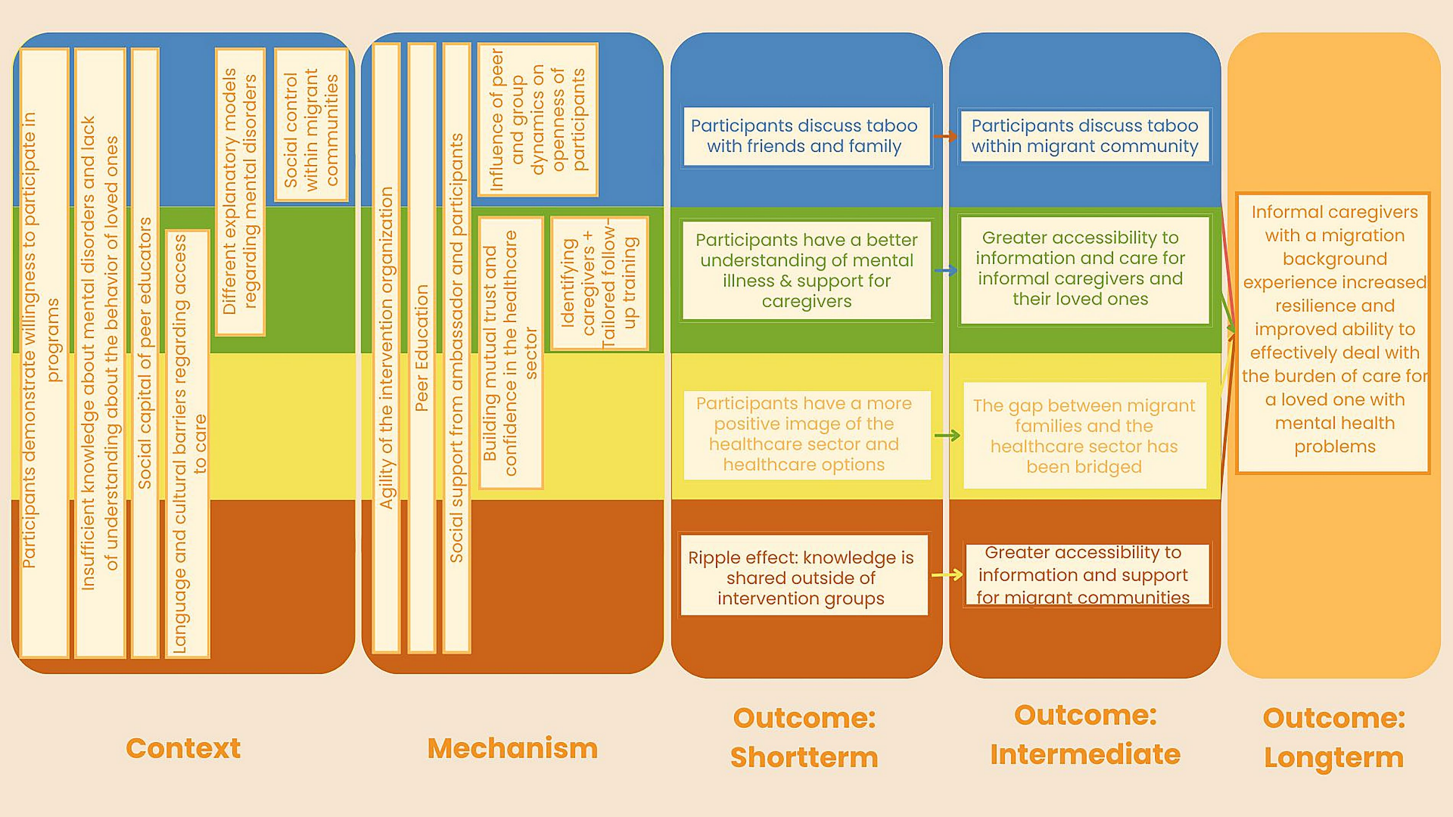
Initial program theory of TANM.

The RE of TANM, combines these methods in four phases. The first phase, which has already been completed (see Figures 2, 3), focused on developing an initial program theory. This theory, presented in section 3.1, serves as the foundation for the remaining phases of our RE. This protocol outlines the steps for phases 2–4, involving: (1) interviews with participants, peer educators, and other stakeholders; (2) post-intervention questionnaires; and (3) observations of the peer education cycle sessions. This study is conducted by a research team with experience in intercultural research, including two researchers who have a migration background themselves. The following sections provide detailed descriptions of each phase and method (see Figure 2).

The study protocol was approved by the Ethics Review Committee of the Department of Psychology, Education and Child Studies, Erasmus University Rotterdam (application number ETH2425-0079).

### 3.1 Phase 1: development of the initial program theory

The first phase of our RE involved developing an initial program theory, which explains which mechanisms caused what outcomes in what contexts (36). Our theory was based on a logic model, derived from available intervention documentation (e.g., ambassador training manual), six interviews with ambassadors and six interviews with caregivers who were part of the 2023 intervention round, and a focus group with the members of the coordinating organizations. Data were collected and analyzed in April–June 2024, and the initial program theory was drafted by one researcher (MH) and refined through iterative discussions with co-authors (PK, SD). The last draft was discussed with the project lead of TANM and then finalized. The initial program theory forms the basis of the further evaluation, for which the methodology is presented in this protocol. The theory is outlined in the next paragraph and in Figure 3.

In our analysis, we found nine CMO-configurations that fitted in four clustered themes, as can be seen in Figure 3. The first theme we found is related to the taboo on mental illness among migrant communities (pictured in blue). The second theme related to increasing accessibility to information, care, and support (pictured in green). The third theme related to bridging the gap between migrant families and the healthcare sector (pictured in yellow). Lastly, the fourth theme related to community empowerment and social capital (pictured in red). The nine CMO-configurations within these four themes serve as a starting point for the data collection and analysis in the following phases of the RE. For more details about our initial theory, see Supplementary material I where we shortly discuss the cluster themes and details of each CMO-configuration.

### 3.2 Phase 2: data collection for theory refinement

Phase 1 resulted in a conceptual model which will guide further empirical data collection aimed at testing and refining it – that is, adapting the program theory based on this new evidence (36). During the most recent intervention cycle, which started in September 2024, researchers will collect qualitative data (i.e., interviews, observations), quantitative data (i.e., post-intervention questionnaire) and logbook data (e.g., notes of meetings with TANM’s coordinating organizations and feedback meetings with peer educators). Recurring consultations with the coordinating organizations were held throughout the development of the research protocol. Data will be collected by a team of co-researchers with diverse cultural backgrounds, including Moroccan, Turkish, Indonesian, and Dutch. This equips the team with insights and skills to connect with participants in a culturally sensitive manner. Supplementary material I elaborates on the initial program theory, while Tables 1, 2 provide an overview of how each CMO-configuration is linked to at least one of the above-mentioned data sources. This ensures that all CMO-configurations are addressed through planned data collection and data analysis. With our approach, we will assess short-term and intermediate outcomes of the TANM intervention components and contextual factors.

**Table 1:** Link between CMO-configurations and data sources.

CMO ID	Context-mechanism-outcome configuration: summary	Data collection method
1a	Ambassadors model openness through sharing personal caregiving and mental illness stories. This reduces stigma and encourages participants to discuss mental health topics and to seek help within and beyond their group.	Interviews, questionnaire, observations
1b	Participants, motivated to understand their loved one’s mental illness, gain emotional and informational support from fellow participants and ambassadors. This fosters discussions reducing stigma and enhancing understanding within and beyond their group.	Interviews, questionnaire, observations
2a	Ambassadors share information and experiences to bridge language and cultural gaps. This helps informal caregivers and migrant families build trust in the Dutch healthcare system and develop more positive perceptions.	Interviews, observations
2b	Interactions with representatives from care organizations during peer education sessions help migrant families overcome distrust and unfamiliarity. These foster increased trust and willingness to seek help from the Dutch healthcare system.	Interviews, observations
3a	Peer education and informational support enhance migrant families’ awareness of available healthcare options. This improves accessibility and fostering better utilization of support services.	Interviews, observations
3b	Ambassadors identify informal caregivers and refer them for tailored follow-up support. This follow-up equips them with the skills and support needed to manage caregiving responsibilities and burdens effectively.	Interviews
4a	Trusted peers (i.e., ambassadors) with strong community ties recruit participants and create a safe, reassuring environment. This increases participation in mental health support programs, despite stigma or mistrust.	Interviews, questionnaire
4b	Participants share knowledge and personal stories with their intervention groups and with their communities. This reduces stigma and spreading understanding about mental health and caregiving.	Interviews, questionnaire, observations
4c	From an organizational perspective, flexibility in adapting training content, communication, and support to cultural and practical needs is needed. This enables inclusive participation and enhances access to information and care for migrant families.	Interviews, observations

**Table 2:** Overview of data collection methods, CMO-configurations, sample size, frequency, and purpose in realist evaluation.

Method	CMO-configuration	Sample size/frequency	Purpose in realist evaluation
Interviews with ambassadors, semi-structured	1a, 1b, 2a, 2b, 3a, 3b, 4a, 4b, 4c	*n* = 10	Understanding ambassador’s perspectives on CMO-relationships
Interviews with participants, semi-structured	1a, 1b, 2a, 2b, 3a, 3b, 4a, 4b, 4c	2 per ambassador, total *n* = 20	Exploring firsthand experiences of short-term outcomes and factors that influenced these changes
Observations	1a, 1b, 2a, 2b, 3a, 4b, 4c	1–2 per group	Capturing real-time behaviors of ambassadors and participants and context
Post-intervention questionnaire	1a, 1b, 4a, 4b	Approx. *n* = 100 (10 ambassadors, ~10 participants each, varying)	Identifying patterns and insights from participants to support interpretation of qualitative findings
Logbook	1a, 1b, 2a, 2b, 3a, 3b, 4a, 4b, 4c	All attended meetings with TANM’s coordinating organizations and all feedback meetings	Providing context for interpreting the findings and aligning them with the program theory

#### 3.2.1 Interviews with ambassadors

The interviews with ambassadors aim to explore their experiences with TANM and will be guided by topics from our initial program theory. This enables exploration of contextual elements and mechanisms driving the intervention’s intended short-term outcomes. The outcomes are related to discussing taboos with friends and family, a better understanding of mental illnesses and caregiver support, a more positive perception of the healthcare system, and the ripple effect where knowledge is shared outside of the intervention group (39).

Table 1 provides an overview of all CMO-configurations, whereas Table 2 provides an overview of which CMO-configurations will be covered in the interviews with the ambassadors.

#### 3.2.2 Interviews with participants

Like the interviews with the ambassadors, those with TANM’s participants aim to explore their experiences with TANM, focusing on how and which intervention components and contextual factors influence TANM’s observed and experienced short-term outcomes. These interviews will address the same CMO-configurations explored in the interviews with the ambassadors (see Tables 1, 2).

We will interview two types of participants; (1) Those identified as informal caregivers and who attended Phase 2, the peer educational cycle, and Phase 3, the tailored follow-up support; and (2) participants who only took part in Phase 2.

#### 3.2.3 Observations

The observations aim to document how the peer educational sessions are delivered, with a focus on contextual factors and mechanisms. We will look at mechanisms, such as providing informational support and discussing individual experiences, and contexts, such as group dynamics and alternative explanatory models of mental illnesses. See Tables 1, 2 for a full overview of the related CMO-configurations.

Observations were conducted using a semi-structured observation guide, designed to capture context and mechanisms from the initial program theory (e.g., social support based on Cohen & Wills (40), aspects of peer education based on Voorham (32)). Specifically, we will focus on:

1. *Social influences*: References to cultural norms, values, and alternative explanations for mental illnesses; individual experiences shared by participants or ambassadors.
2. *Social support*: Types of social support observed and the ways in which they are expressed.
3. *Role of the ambassador*: Adaptations of content by the ambassador (e.g., translation or cultural tailoring); peer education aspects such as similarity and professionalism.
4. *Group dynamics*: Interactions, trust, inclusivity, dominance of specific members, and both verbal and nonverbal communication.

The guide includes space for additional notes and researcher reflections after observations (full guide is added in Supplementary material II).

The observations will take place during Phase 2 of TANM, with at least one session per intervention group observed by researchers who are fluent in the language spoken in each group. This ensures an accurate understanding of what is observed. Given the expected sensitivity of the topic and the potential caution of participants toward outsiders like researchers, we made efforts to minimize interference with the natural flow of the educational sessions. Accordingly, we schedule our observations to coincide with planned visits from TANM’s coordinating organizations’ representatives. As such, we observe all final sessions, as we are present to administer the questionnaire (see 3.2.4). Additionally, if an organizational visit is planned during the first or second peer education session, we join these visits, observing these sessions as well. This will total to at least 10 observations from the final peer education session, added by one to 10 observations from the first or second sessions. This will result in at least 10 observations of the final peer education sessions, with an additional one to 10 observations from the first or second sessions.

#### 3.2.4 Questionnaire

The quantitative component of this study is of exploratory nature and involves the use of a post-intervention questionnaire conducted among participants of the peer education sessions. It aims to capture differences between participant groups, and it will collect insights into the context and mechanisms related to social support, perceived stigma, group composition, and group dynamics (see Tables 1, 2).

All Phase 2 participants are asked to complete the questionnaire at the end of the final third session. Based on advice of the coordinating organizations, we address practical challenges associated with the target group, such as expected language barriers, diverse backgrounds, and varying literacy levels, the questionnaire was simplified to a maximum B2-level in Dutch. We minimized the number of questions and made answer options straightforward (e.g., yes/sometimes/no) to avoid confusion.

At the end of the third session, the attending researcher distributes paper questionnaires and explains the study’s goal, questionnaire, and informed consent, and instructs participants to complete them individually. In groups where participants do not understand Dutch, a research assistant fluent in the group’s language translates each question aloud, one at a time, for the entire group. This approach allows participants to listen to the translation and fill in their responses individually.

Rather than relying on pre-validated questionnaires, the items were constructed based on theoretical concepts from our CMO-configurations. The questionnaire starts with demographic questions (i.e., age, gender, country of birth of the participant and their parents), and questions related to caregiving responsibilities (i.e., frequency and care recipient). It is followed by section on group composition (specifying familiarity and relationships with group members and ambassador prior to the intervention), social support mechanisms (inspired by Cohen and Wills (40)), and mental health taboos and group dynamics. Lastly, an open-ended section allows participants to share lessons or insights gained during the intervention. Table 3 provides an overview of the themes and items, with the full questionnaire available in Supplementary material III.

**Table 3:** Overview of questionnaire themes, items and example questions.

Questionnaire themes	Number of items	Examples of items and answer options
Group composition	5	Did you know the other participants before the start of the sessions? Answer categories: yes/no. If yes, where from? Answer categories: previous training, church, (volunteer) work, common friends or acquaintances, neighborhood or district.
Social support mechanisms	5	Can you talk to the other group members when you do not feel well or need support? Answer categories: yes/sometimes/no
Mental health taboos and group dynamics	4	Did your group members talk about their experiences with caring for a loved one? Answer categories: yes/sometimes/no

#### 3.2.5 Logbook

To document the study process and decisions made throughout, researcher MH makes notes in a logbook. This logbook contains notes of all attended meetings with TANM’s coordinating organizations as well as feedback meetings held with the ambassadors and representatives from the coordinating organizations. The notes capture key decisions during the study and intervention process, feedback received by the coordinating organizations and ambassadors, and personal reflections of the researcher. As such, these notes will provide context for interpreting the findings and aligning them with the program theory, contributing to a nuanced interpretation of the results and supporting the data triangulation process in Phase 3. The template of the logbook has been added to Supplementary material IV.

### 3.3 Phase 3: data analysis and theory refinement

The analysis process comprises method-specific analyses for each type of data collected, followed by an integrative synthesis to triangulate our findings and refine the initial program theory. Below, we describe each type of analyses, followed by a description of how we will triangulate the data and refine the program theory.

#### 3.3.1 Qualitative data: interviews and observations

Audio recordings of interviews will be transcribed verbatim, and observation schemes, completed during fieldwork, will be digitalized. Additionally, the open-ended question from the post-intervention questionnaire will be transcribed and analyzed as part of the qualitative data.

Thematic analysis will be conducted using both deductive and inductive approaches. Deductive analysis is guided by the initial program theory, with a coding framework structured around the concepts within our CMO-configurations (see Figure 3). At the same time, inductive analysis allows new themes and mechanisms to emerge within the predefined categories of context, mechanisms, and outcomes (41).

The analysis will follow Braun and Clarke’s (42) six-step framework: starting with familiarization with the data through repeated reading, followed by systemic coding, theme development and refinement. Coding will be conducted in Atlas.ti [Version 24; (43)].

#### 3.3.2 Quantitative data: questionnaire

Quantitative data from the post-intervention questionnaire will be analyzed using SPSS [Version 29.0.0.0; (44)] to examine differences across intervention groups and assess the potential influence of contextual factors on perceived mechanisms and outcomes. Based on the initial program theory, we expect differences between groups (i.e., whether participants knew each other or their ambassador beforehand and the cultural homogeneity of the group), which may influence social support and group dynamics (such as sharing personal experiences with caregiving and/or mental illness and perceptions of taboo). To explore these potential mechanisms within our program theory, we will conduct crosstabulations of these independent and dependent variables, supplemented by chi-square tests to assess the statistical significance of observed differences.

Based on previous studies on peer education with migrants [e.g., (45, 46)], we estimate an effect size of 0.4. Using a power analysis in R (47) with the pwr package (48) we expect to need around 60 completed surveys to achieve sufficient power. Given that we anticipate collecting around 8–12 questionnaires per intervention group, totalling approximately 80–100 questionnaires, we expect to have adequate power for our questionnaire analysis.

Missing data will be included in the analyses as is, unless responses to other questions suggest that the omission can be reasonably filled in based on available information. In such cases, missing responses may be assessed using other relevant data from the same participant. All decisions regarding missing data will be reported transparently in the final analysis.

#### 3.3.3 Data triangulation

The data triangulation process will integrate findings from the interviews, observations, and post-intervention questionnaires. Firstly, we will identify patterns where different findings from different data sources align, confirming the validity of specific CMO-configurations from our initial program theory. Discrepancies will be explored, by analyzing the inconsistencies between data sources to understand the variations in context, mechanisms, or outcomes.

The logbook that was kept by researcher MH will serve as a tool to help identify explanations of discrepancies between sources, by offering rationale behind actions and decisions, as well as personal reflections of the study process.

Inspired by Rees (49), a synthesis matrix will be developed to systematically map findings from all sources to the initial CMO-configurations. This matrix will be organized around the four overarching themes from our initial program theory (See Figure 3). Under each theme, the relevant CMO-configurations will be listed, along with associated concepts from the program theory. Data from the interviews, observations and post-intervention questionnaires will be aligned with these themes, allowing for identification of new or modified CMO-configurations, where applicable. This will ensure a structured approach to integrate qualitative and quantitative findings and provide an overview of how data supports or challenges the initial program theory.

#### 3.3.4 Refinement of the program theory

Insights gained from the integrated data will guide the iterative refinement of the program theory. This process starts with evaluating the existing CMO-configurations of the initial program theory based on the triangulated evidence. If the findings indicate that certain configurations are only partially supported or not confirmed, they will be adjusted accordingly. Additionally, if the data reveals previously unconsidered mechanisms or contextual factors, new CMO-configurations will be developed and integrated into the program theory.

The first author (MH) leads the refinement process, while the co-authors provide feedback and discuss discrepancies. We will mitigate potential bias caused by our positionalities – i.e., our backgrounds and perspectives - through regular discussions within the research team. Additionally, throughout the project, regular meetings are held with the TANM’s coordinating organizations to discuss the progress and research design, review the preliminary results, and adjust accordingly.

### 3.4 Phase 4: final validation of the program theory and evaluative synthesis

The adjusted program theory (Phase 3) will be subjected to a validation process to ensure its relevance and credibility. The theory will be validated in a focus group with the members of t TANM’s coordinating organizations. Feedback and information received during the focus group could lead to one last round of refinement, before reaching the final program theory of TANM. Once the program theory is finalized, we can answer our research questions concerning the perceptions of a successful change in TANM’s short-term outcomes. The process is concluded with an evaluative synthesis of TANM, that assesses whether it achieves it outcomes and which factors influence its success.

## 4 Discussion

While previous studies have explored experiences of informal caregivers with a migration background [e.g., (50, 51)] and the stigma surrounding mental illness within migrant communities [e.g., (52–54)], this study will build on that foundation by evaluating an intervention that is aimed at supporting informal caregivers with a migration background who care for a loved one with a mental illness. This protocol shows how we plan to investigate how, why, for whom and under what circumstances TANM produces its intended outcomes.

This protocol employs a RE approach, which is suitable for evaluating interventions that are dynamic, culturally sensitive and context-dependent – like TANM. Unlike traditional randomized experiments, which may not capture the complexity or nuances of real-world settings, RE allows for the consideration of variations in context and experiences as integral to understanding outcomes (27, 55). Its flexibility helps to overcome challenges such as language barriers (35). This makes it a fitting approach for evaluating culturally sensitive interventions like TANM. RE extend qualitative insights to broader patterns by linking context, mechanisms and outcomes, making it more useful for informing policy and practice (27, 55) – which is ultimately the goal of our evaluation of TANM.

However, the flexibility of RE also presents challenges, such as the lack of clear methodological guidance (56). To address this, we employed a mixed-methods approach (35) and multiple data sources for triangulation. Additionally, while RE often uses arrows within program theories, they do not always establish universal causality. Depending on the selection of methods applied in a RE, causality is not necessarily implied. Instead of aiming for universally applicable explanations, REs develop so-called “middle-range” theory – explanations that are specific enough to be useful in practice but broad enough to apply beyond a single case. These theories link empirical findings to hypotheses about how an intervention works, helping to guide future research and program implementation, rather than providing definitive conclusions (56). This study’s program theory will serve as a foundation for future research on culturally sensitive caregiver interventions, such as longitudinal studies assessing long-term impacts or comparative research across different cultural contexts to explore their influence on outcomes.

Our RE approach aligns with its growing use in health system research, particularly in transdisciplinary collaborations. Similar to previous studies [e.g., (57, 58)] we primarily employ qualitative and exploratory methods to evaluate TANM. By sharing the lessons learned from implementing this protocol, we aim to inform the design and evaluation of culturally tailored interventions in integrated healthcare, particularly in reaching underserved and hard-to-reach populations (5, 20).

### 4.1 Strengths and limitations

A key strength of this study is its use of multiple data sources for triangulation, adapted to participants’ cultural, linguistic and health literacy needs. Additionally, through collaboration with TANM’s coordinating organizations and ambassadors, the relevance and approach of our study is enhanced by including people with lived experiences of integrated care. We expect to have a suitable approach in our research. However, as an exploratory study, it cannot establish causality but rather provide insights for future research and practice (56).

Conducting research alongside intervention implementation also required pragmatic decisions. For instance, the post-intervention questionnaire was only available in Dutch, though spoken translations were provided in Turkish, Arabic, and Tamazight, when possible, either by a researcher or a bilingual participant willing to help. Additionally, recall bias may affect interviews conducted after the intervention. We mitigated this through real-time observations, which provided immediate insights to cross-verify participant accounts.

Cultural and language differences may also introduce biases, such as translation bias, where meaning is lost or altered when translating questions or responses (59); and interpretation bias, where differences in cultural norms or expression lead to the wrong interpretation of participants’ response (59, 60). While efforts were made to minimize these biases through matching researchers with participants (e.g., Turkish-speaking researchers for Turkish-speaking groups), this could either facilitate openness or lead participants to withhold information due to feelings of shame or stigma when the researcher was perceived as an insider of their community (61). To navigate these complexities, we incorporated team reflections to account for cultural nuances and researcher positionality.

## 5 Conclusion

This protocol outlines our approach for evaluating and refining TANM’s program theory. By integrating multiple data collection methods and applying both inductive and deductive analyses, we aim to understand the intervention’s mechanisms and whether participants, ambassadors, and coordinators perceive improvements in discussing mental health taboos, understanding mental illnesses and caregiver support, and fostering trust in the healthcare system. Beyond TANM, this study contributes to broader research, policy, and practice in integrated care, particularly in reaching vulnerable populations, including those with a migration background.
