# Supplementary material for: Exploring peer education for migrant informal caregivers of mentally ill loved ones: a realist evaluation protocol
Source: Front Public Health. 2025 Aug 13;13:1623903. doi: 10.3389/fpubh.2025.1623903 (PMC12380533; doi:10.3389/fpubh.2025.1623903)
Supplement: Supplementary file 1 [file Data_Sheet_1.docx]

## Appendix I – Contents of each peer education session

|  | **Topic** | **Aims** |
| --- | --- | --- |
| **Session 1** | Severe mental health issues and the role of culture | - Ambassadors gain insight into how participants make psychiatric issues discussable and no longer perceive them as a taboo; - Participants enhance their knowledge about the origins of mental health problems; - Participants are aware that they can seek help early on; - Participants share their experiences regarding their perception of the illness, such as heredity and ‘being possessed’. |
| **Session 2** | Mental health issues: psychosis, schizophrenia and depression | - Participants receive basic information about depression, psychosis, and schizophrenia; - Participants share their experiences with the group; - Participants know where they can potentially get help for psychiatric issues and addiction; - Participants have insight into treatment option; - Participants are motivated to seek support, both for the patient and the caregiver; - Caregivers know where they can turn for assistance. |
| **Session 3** | The family: what can the family do, and what support is available for the family | - Participants may meet experts and receive information about caring for an ill family member; - Participants learn about their legal position and guidance options; - Participants have the opportunity to ask questions; - Discuss differences in caregiving beliefs and communication; - Explain the referral process within healthcare; - Promote better, open communication and trust between migrant families and healthcare institutions. |
