# Supplementary material for: Exploring peer education for migrant informal caregivers of mentally ill loved ones: a realist evaluation protocol
Source: Front Public Health. 2025 Aug 13;13:1623903. doi: 10.3389/fpubh.2025.1623903 (PMC12380533; doi:10.3389/fpubh.2025.1623903)
Supplement: Supplementary file 2 [file Supplementary_file_1.docx]

Supplementary Material 1

In our analysis, we found nine CMO-configuration who fit within four clustered themes, as can be seen in Figure 2. First, we found configurations related to the taboo on mental illness within migrant communities. Second, we found the configurations related to bridging the gap between migrant families and the healthcare sector. Third, we found configurations related to increasing accessibility to information and care and support. Lastly, we found configuration related to community empowerment and social capital. These nine CMO-configurations serve as a starting point for the data collection and analysis in the following phases of the RE. In this appendix, we shortly discuss the cluster themes and detail each CMO-configuration.

####
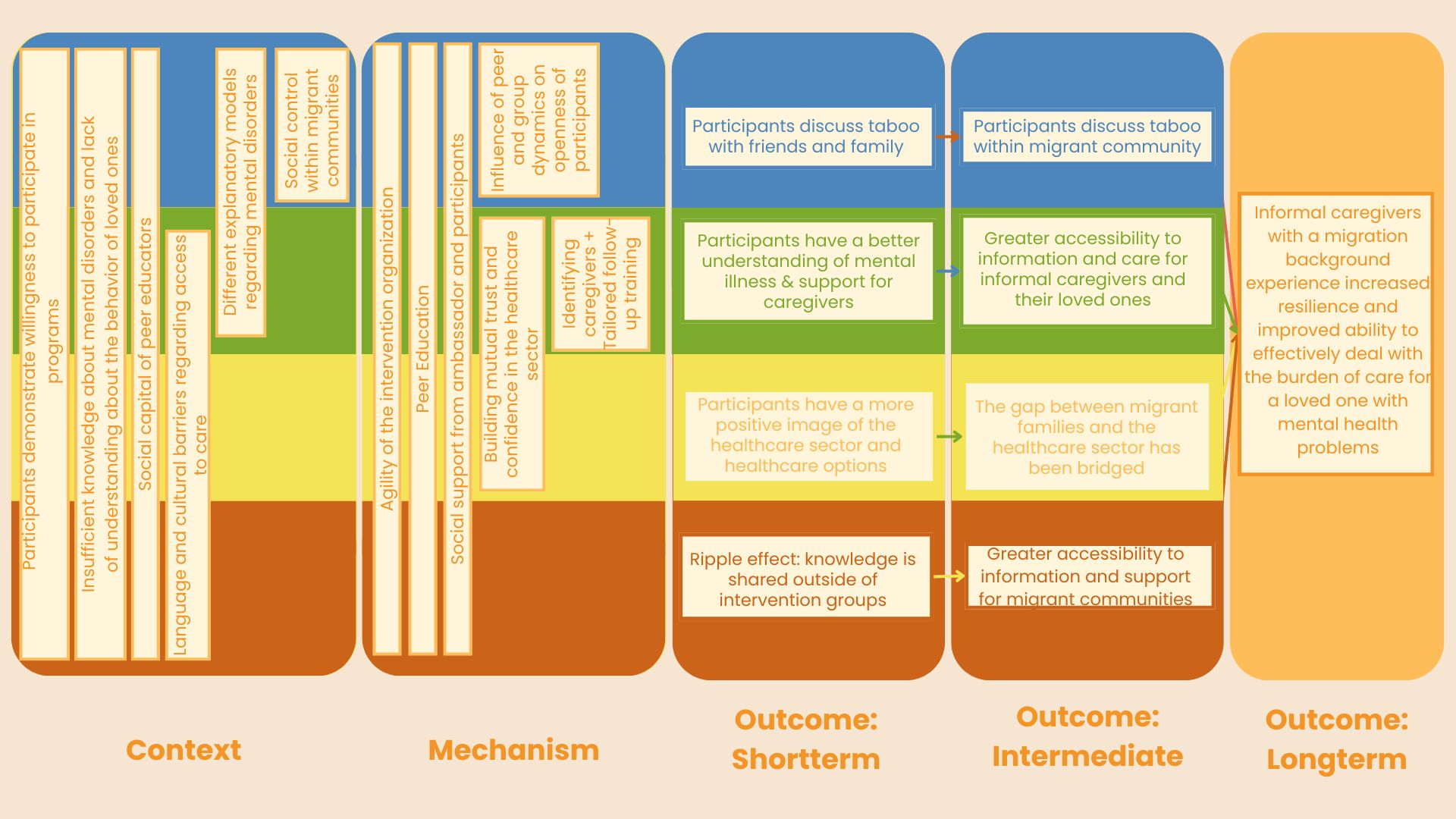


#### Cluster 1: Discussing the taboo on mental health

In close-knit migrant communities, where diverse explanatory models for mental health conditions are common and fear of gossip often prevails, informal caregivers may feel unable or unwilling to speak about their situations and are thus less likely to seek help. In order to address this problem, peer education and social support encourages discussions on the taboo surrounding mental illness and seeking support.

- **CMO 1a: Peer Education and Modeling**

**Context:** Migrant communities often have diverse beliefs and explanations for mental illness. The subsequent feelings of shame and fear of gossip, discourages caregivers from discussing their situation and prevents them from seeking help.

**Mechanism:** Ambassadors share personal stories of caregiving and mental illnesses, modeling openness for participants and encouraging them to share their experiences with others.

**Outcome:** Topics of mental illness, seeking professional help and caregiving are discussed within the intervention group and outside of the group, with friends and families.

- **CMO 1b: Improved Understanding**

**Context:** Participants need to be willing to attend several sessions of a course, and they are often motivated by a wish to improve their understandings of the behavior of their loved one with a mental illness.

**Mechanism:** Emotional and informational support from both the ambassador and fellow group members improves the understanding of mental illnesses and the role of family

**Outcome:** Increased understanding will enable further discussion around the stigma and shame surrounding mental health issues within and outside of the group.

#### Cluster 2: Bridging the gap between migrant families and the healthcare sector

In a context where migrant families experience a disconnect with the Dutch healthcare system due to language and cultural barriers—leading to unfamiliarity with and negative perceptions of the healthcare system—providing information about healthcare services and available support options can help foster a more positive view of the system. This information is shared through peer education, where facilitators with a migration background share their own caregiving experiences and positive interactions with the healthcare sector. Furthermore, encounters with members from the organization of the intervention (who represent different health care organizations) help to build trust in the healthcare system.

- **CMO 2a: Peer education and Accessibility**

**Context:** Migrant families often face language and cultural barriers that create a disconnect with the Dutch healthcare system.

**Mechanism:** Ambassadors share information and positive experiences with the healthcare system and caregiving.

**Outcome:** Caregivers develop a more positive perception of the healthcare system.

- **CMO 2b: Creating Trust Through Meetings with Care Providers**

**Context:** Migrant families’ disconnect with the Dutch healthcare system is caused by unfamiliarity and distrust in the system.

**Mechanism:** Members from the intervention’s organization each represent different (health) care organizations. They visit at least one of the three peer education sessions per group, so participants meet and interact with representatives from care organizations in order to build trust.

**Outcome:** Increased trust in the Dutch healthcare system and willingness to seek help.

#### Cluster 3: Improved access to information and support

Within the intervention, participants’ willingness to attend courses, driven by a desire to better understand their loved ones’ behavior, motivates engagement in informational sessions and optional follow-up training. These sessions, delivered through peer education and informational support, provide guidance from the ambassadors and foster the exchange of mutual advice among participants, enhancing knowledge of mental health conditions, cultural influences, family roles, and available support options. In the context of migrant families, who often face language and cultural barriers and limited familiarity with the Dutch healthcare system, this approach improves access to care and increases caregivers’ willingness to seek help. By bridging gaps in knowledge and support, the intervention strengthens caregivers’ ability to manage their responsibilities.

**CMO 3a: Increased Accessibility Through Peer Education**

**Context:** Migrant families are less familiar with the available support options within the Dutch healthcare system.

**Mechanism:** Peer education and informational support offer guidance on available care and support options.

**Outcome:** Increased accessibility to healthcare and support options for migrant families.

- **CMO 3b: Support with Tailored Follow-Up Training**

**Context:** Some participants might be informal caregivers who need additional support.

**Mechanism:** Ambassadors identify informal caregivers within their group of participants and refer them to the organization. Tailored follow-up training is provided for those with additional needs.

**Outcome:** Caregivers are better equipped to manage caregiving responsibilities and cope with caregiving burdens.

#### Cluster 4: Community empowerment through social capital

Community empowerment and social capital focus on the process of leveraging social networks and trust within migrant communities, to achieve a broader impact. Ambassadors (i.e. the peer educators) play a central role, they have built op social capital through long-term involvement in their communities and act as trusted community figures. Using their networks, they are able to recruit participants who might otherwise not have become involved with the intervention. In turn, the intervention encourages participants to share the knowledge they gained and their experiences outside of the educational sessions, e.g. with their friends and families. This raises awareness within the community about mental health and informal caregiving, which leads to a wider outreach to informal caregivers who feel supported and might be more willing to seek help.

- **CMO 4a: Recruitment Through Trusted Community Figures**

**Context:** Migrant communities may be hesitant to engage in mental health support programs due to stigma, mistrust or feelings of shame.

**Mechanism:** Ambassadors who are trusted community figures with established social capital, can leverage their relationships to recruit participants and reassure participants about the intervention being a safe environment.

**Outcome:** Increased participation in the intervention as community members feel more comfortable attending when they are approached by someone they know and trust.

- **CMO 4b: Participants sharing knowledge beyond the group**

**Context:** Migrant communities have diverse beliefs, understandings and explanations for mental illness, which causes stigma and misunderstandings.

**Mechanism:** Participants share knowledge and personal stories within their groups and are encouraged by their ambassadors to keep talking about these topics outside of the group, with friends and family.

**Outcome:** Knowledge about mental health and caregiving is shared, creating a ripple effect across migrant communities.

- **CMO 4c: Organizational Flexibility**

**Context:** Reaching hard-to-reach migrant families often require tailored approaches that address their cultural and practical needs.

**Mechanism:** Flexibility of the organization enables the adaptation of training content, communication and follow-up support to meet the specific needs of participants, as well as needs of the ambassadors.

**Outcome:** More inclusive participation of migrant communities and greater accessibility of information and care for informal caregivers.
