# Supplementary material for: Exploring peer education for migrant informal caregivers of mentally ill loved ones: a realist evaluation protocol
Source: Front Public Health. 2025 Aug 13;13:1623903. doi: 10.3389/fpubh.2025.1623903 (PMC12380533; doi:10.3389/fpubh.2025.1623903)
Supplement: Supplementary file 3 [file Supplementary_file_2.docx]

# Supplementary Material II: Observatieschema: ZZTNG?!

Datum & Tijd:

Locatie (e.g. buurthuis, bibliotheek, etc):

Welke bijeenkomst (1, 2 of 3):

Naam Observator:

| **Sociale Invloed** | | | |
| --- | --- | --- | --- |
| ***Instructie: benoem niet alleen hoe vaak het ter sprake komt, maar ook bij hoeveel mensen (b.v. als maar twee mensen actief mee doen, dan is er minder spreiding over hele groep)*** | | | |
| **Wordt er gerefereerd aan hun cultuur? / Wordt er gesproken over:** | | | |
| Normen en waarden | | 1 2 3 4 5 | |
| Geloof | | 1 2 3 4 5 | |
| Woorden gekoppeld aan geneeswijzen, verklaringsmodellen | | 1 2 3 4 5 | |
| **Worden er persoonlijke ervaringen en verhalen gedeeld?** | | | |
| Ambassadeur | | 1 2 3 4 5 | |
| Deelnemers | | 1 2 3 4 5 | |
| Details/Voorbeelden | | | |
|  | | | |
| Notities (b.v. onderwerpen van de verhalen, door wie, hoe reageren anderen?) | | | |
|  | | | |
| **Sociale Steun** | | | |
| ***Instructie: benoem niet alleen hoe vaak het ter sprake komt, maar ook bij hoeveel mensen (b.v. als maar twee mensen actief mee doen, dan is er minder spreiding over hele groep)*** | | | |
| Welke types sociale steun worden er waargenomen? | | | |
| Emotioneel | b.v. troost, aanmoediging | |  |
| Informationeel | b.v. advies, informatie delen | |  |
| Instrumenteel | b.v. hulp bij praktische zaken | |  |
| Sociaal gezelschap | b.v. samen een activiteit, afleiding van het onderwerp | |  |
| Details/Voorbeelden | | | |
|  | | | |
| Notities (b.v. over interacties die de types sociale steun illustreren) | | | |
|  | | | |

| **Rol van de ambassadeur** | | | | |
| --- | --- | --- | --- | --- |
| Wordt de inhoud aangepast door de ambassadeur? | | | | |
| Similariteit | Gelijkheid en gemeenschappelijk belang met de groep, zoals sekse, leeftijd, etniciteit, migrant zijn, lotgenoot, etc. | | 1 2 3 4 5 | |
| Professionaliteit | Mate van professionaliteit van de *peer* | | 1 2 3 4 5 | |
| Taalniveau wordt vereenvoudigd | b.v. vereenvoudigd moeilijke woorden, herhaalt belangrijke punten | | 1 2 3 4 5 | |
| Vertalingen | b.v. losse termen of hele zinnen worden vertaald naar andere taal | | 1 2 3 4 5 | |
| Culturele Aanpassingen | b.v. vermijd bepaalde onderwerpen of bewoordingen, gebruikt lokale spreekwoorden | | 1 2 3 4 5 | |
| Details/Voorbeelden | | | | |
|  | | | | |
| Notities (b.v. watvoor aanpassingen? Geven ze ook aan waarom? *niet zelf teveel interpreteren!) | | | | |
|  | | | | |
| **Groepsdynamiek** | | | | |
| Hoe is de interactie binnen de groep? | | | | |
| Vertrouwen en openheid | | b.v. open gesprekken, voorzichtigheid | |  |
| Dominantie deelnemers | | b.v. iemand neemt veel de leiding | |  |
| Inclusiviteit | | b.v. iedereen wordt betrokken bij het gesprek | |  |
| Verbale communicatie, non-verbale communicatie | | b.v. houding ten opzichte van de groep | |  |
| Details/Voorbeelden | | | | |
|  | | | | |
| Notities (b.v. groepsgedrag, interacties of andere opvallende dynamieken?) | | | | |
|  | | | | |

| **Overig** |
| --- |
| Beschrijf andere opvallende momenten of gedragingen die je hebt waargenomen |
|  |

| **Na afloop reflectie** |
| --- |
| Ruimte voor de observator om algemene indrukken en overkoepelende gedachten over de bijeenkomst te delen |
|  |
