# Supplementary material for: Exploring peer education for migrant informal caregivers of mentally ill loved ones: a realist evaluation protocol
Source: Front Public Health. 2025 Aug 13;13:1623903. doi: 10.3389/fpubh.2025.1623903 (PMC12380533; doi:10.3389/fpubh.2025.1623903)
Supplement: Supplementary file 4 [file Supplementary_file_3.docx]

## Supplementary Material III: Vragenlijst Groepsbijeenkomst ‘Ze Zijn Toch Niet Gek?’

| **Algemene Gegevens** | | |
| --- | --- | --- |
| **Hoe oud ben je?** |  | |
| **Ik ben een** | - Man - Vrouw | |
| **In welk land ben jij geboren?** | - Nederland - Turkije - Marokko - Suriname - Nederlandse Cariben - Indonesië - Syrië - Anders, namelijk: _________________________ | |
| **In welk land is jouw moeder geboren?** | - Nederland - Turkije - Marokko - Suriname - Nederlandse Cariben - Indonesië - Syrië - Anders, namelijk: _________________________ | |
| **In welk land is jouw vader geboren?** | - Nederland - Turkije - Marokko - Suriname - Nederlandse Cariben - Indonesië - Syrië - Anders, namelijk: _________________________ | |
| **Zorg jij voor iemand die ziek is of extra hulp nodig heeft?** | - Ja, elke dag - Ja, elke week en minder dan elke dag - Ja, elke maand en minder dan elke week - Nee | |
| Zo ja, voor wie zorg jij? | - Vader of moeder - Broer of zus - Zoon of dochter - Buurvrouw of buurman - Anders, namelijk:   ______________________________ | |
| Over de groep | | |
| **Woon je in dezelfde buurt als de andere groepsleden?** | | - Ja - Nee - Weet ik niet |
| **Kende je de andere groepsleden al voordat de bijeenkomsten begonnen?** | | - Ja - Nee |
| Zo ja, waar kende je de groepsleden van? | | - Eerdere cursus - Kerk - Werk of vrijwilligerswerk - Gemeenschappelijke vrienden of kennissen - Wijk of buurt |
| **Kende je de ambassadeur al voordat de bijeenkomsten begonnen?** | | - Ja - Nee |
| Zo ja, waar kende je de ambassadeur van? | | - Eerdere cursus - Kerk - Werk of vrijwilligerswerk - Gemeenschappelijke vrienden of kennissen - Wijk of buurt |
| **Voel je je gesteund door de andere groepsleden?** | | - Ja - Soms - Nee |
| **Krijg je hulp van de andere groepsleden, bijvoorbeeld met praktische klusjes of taken?** | | - Ja - Soms - Nee |
| **Kan je met de andere groepsleden praten als je je niet goed voelt of steun nodig hebt?** | | - Ja - Soms - Nee |
| **Voel je je op je gemak bij de andere groepsleden, bijvoorbeeld door samen te praten of activiteiten te doen?** | | - Ja - Soms - Nee |
| **Heb je nuttige adviezen of informatie gekregen van de andere groepsleden?** | | - Ja - Soms - Nee |
| **Heb je zelf nuttige adviezen of informatie kunnen geven aan andere groepsleden?** | | - Ja - Soms - Nee |

| Over de bijeenkomsten | |
| --- | --- |
| **Voel je dat er een taboe is om over mentale gezondheid te praten?** | - Ja - Soms - Nee |
| **Vind je het moeilijk om over de mentale gezondheid van degene voor wie jij zorgt te praten?** | - Ja - Soms - Nee |
| **Heeft de ambassadeur verteld over haar eigen ervaringen met zorgen voor een ander?** | - Ja - Soms - Nee |
| **Hebben jouw groepsgenoten verteld over hun ervaringen met zorgen voor een ander?** | - Ja - Soms - Nee |
| **Kon jij vertellen over jouw ervaringen met het zorgen voor een ander?** | - Ja - Soms - Nee |
| **Wat zijn de belangrijkste lessen die jij hebt geleerd tijdens de bijeenkomsten?­­­­­­­** | |
| **_______________________________________________________________________________________________________________________________________________________________________________________________________________________________________________________________________________________________________________________________________________________________________________________________________________________________________________________________________________________________________________________________________________________________________________________________________________________________________________________________________________________________________________________________________­_______________________________________________________________________________** | |
