# Supplementary material for: Exploring peer education for migrant informal caregivers of mentally ill loved ones: a realist evaluation protocol
Source: Front Public Health. 2025 Aug 13;13:1623903. doi: 10.3389/fpubh.2025.1623903 (PMC12380533; doi:10.3389/fpubh.2025.1623903)
Supplement: Supplementary file 5 [file Supplementary_file_4.docx]

# Supplementary Material IV: Template Logbook ZZTNG?! / TANM

## Discussiepunten en vragen om te beantwoorden per meeting

1. **Samenvatting van de meeting**

Belangrijkste punten die tijdens de vergaderingen met opdrachtgever en/of onderzoeksteam zijn besproken. Inclusief genomen beslissingen en eventuele actiepunten.

1. **Reflecties op beslissingen**

Beschrijf kort eigen denkproces en redenering achter de genomen beslissingen. Denk na over mogelijke implicaties van deze beslissingen en of ze wel/niet overeenkomen met de doelstellingen van het project.

1. **Gevonden uitdagingen**

Beschrijf eventuele of obstakels die zich tijdens de meeting of het onderzoek hebben voorgedaan. Reflecteer hierop: waarom zijn ze ontstaan en hoe kunnen ze overwonnen worden?

1. **Feedback en suggesties**

Beschrijf eventuele feedback of suggesties die door opdrachtgever of teamgenoten zijn gegeven. Reflecteer op de geldigheid van deze suggesties en hoe ze kunnen bijdragen aan het algehele succes van het onderzoeksproject.

1. **Vragen voor verdere verkenning**

Zijn er nog vragen die ontstaan zijn naar aanleiding van de vergadering? Schrijf ze op voor verdere verkenning of discussie samen met onderzoeksteam en/of opdrachtgever.

1. **Interpretatie van veerkracht**

Op welke manier wordt er door de betrokkenen over veerkracht gesproken? Eventuele andere concepten zoals gezondheid en caregiver burden?

| **Datum** | | **Meeting** | **Aanwezig: … Afgemeld**: … | |
| --- | --- | --- | --- | --- |
| **Locatie:** | | | | |
| 1. **Samenvatting van de meeting** | | *Belangrijkste punten die tijdens de vergaderingen met opdrachtgever en/of onderzoeksteam zijn besproken. Inclusief genomen beslissingen en eventuele actiepunten* | | |
|  | | | | |
| 1. **Reflecties op beslissingen** | | *Beschrijf kort eigen denkproces en redenering achter de genomen beslissingen. Denk na over mogelijke implicaties van deze beslissingen en of ze wel/niet overeenkomen met de doelstellingen van het project.* | | |
|  | | | | |
| 1. **Gevonden uitdagingen** | | *Beschrijf eventuele of obstakels die zich tijdens de meeting of het onderzoek hebben voorgedaan. Reflecteer hierop: waarom zijn ze ontstaan en hoe kunnen ze overwonnen worden?* | | |
|  | | | | |
| 1. **Feedback en Suggesties** | | *Beschrijf eventuele feedback of suggesties die door opdrachtgever of teamgenoten zijn gegeven. Reflecteer op de geldigheid van deze suggesties en hoe ze kunnen bijdragen aan het algehele succes van het onderzoeksproject.* | | |
|  | | | | |
| 1. **Vragen voor verdere verkenning** | | *Zijn er nog vragen die ontstaan zijn naar aanleiding van de vergadering? Schrijf ze op voor verdere verkenning of discussie samen met onderzoeksteam en/of opdrachtgever.* | | |
|  | | | | |
| 1. **Interpretatie veerkracht** | | *Op welke manier wordt er door de betrokkenen over veerkracht gesproken? Eventuele andere concepten zoals gezondheid, caregiver burden?* | | |
|  | | | | |
